# Sexual Embodiment and Sexual Renegotiation Post-Cancer for LGBTQ People with a Cervix

**DOI:** 10.1007/s10508-025-03090-w

**Published:** 2025-03-12

**Authors:** Sam Sperring, Jane Ussher, Rosalie Power, Alexandra Hawkey

**Affiliations:** Translational Health Research Institute, Western Sydney University, Locked Bag 1797, Penrith, NSW 2751 Australia

**Keywords:** LGBTQ, Non-binary, Cancer, Sexuality, Health-care professional, Sexual renegotiation

## Abstract

Lesbian, gay, bisexual, transgender, and queer (LGBTQ) people face unique challenges in the context of cancer due to cis-heterocentric constructions of sexuality in oncological care. This paper explores the impacts of these challenges for LGBTQ people with a cervix, examining embodied sexual changes and sexual renegotiation during and after cancer, and the implications for LGBTQ people’s access to relevant, tailored cancer information and support. Semi-structured interviews were conducted with 57 LGBTQ people with a cervix with cancer and 14 intimate partners, representing a range of cancer types and stages. Data were analyzed using reflexive thematic analysis. Several participants positioned changes to their sexual embodiment as abject, instilling a loss of control and uncertainty. Sexual practices were misunderstood by many health-care professionals (HCPs), meaning concerns about bodily changes, sex, and intimacy were poorly addressed in cancer support resources and medical interactions. This left LGBTQ participants and their partners ill-equipped to cope with changes to sexual functioning, feeling unsupported, distressed, and as though their sexual concerns were of less importance than those of cis-heterosexual patients. However, some LGBTQ participants and partners were able to renegotiate sexual practices through engaging in non-genital intimacy and experimenting with non-penetrative sex. This was facilitated through good couple communication, where needs were openly discussed between partners. Assumptions of heterosexuality and cisgender embodiment, identity, and sexual expression have serious implications for LGBTQ people’s physical and emotional well-being, agency, and sexual health decision-making. These assumptions also affect access to relevant and culturally safe sexual health information and care. Findings reinforce the need for the inclusion of LGBTQ content in HCP education and professional training curricula, and institutional support for LGBTQ-inclusive practice behaviors.

## Introduction

Sexuality and intimacy are crucial for mental well-being and overall life satisfaction (WHOQOL Group, 1995). Cancer and its treatments frequently cause significant changes in sexual health, sexual functioning, and intimate relationships (Gilbert et al., 2010b; Perz et al., 2014; Ussher et al., 2013), making these issues some of the most difficult to manage after cancer (Anderson et al., 2000). Research on women’s sexual function and embodiment after cancer treatment has identified common physical changes to be fatigue, vaginal dryness, decreased libido, and difficulties with achieving orgasm (Mejía-Rojas et al., 2020). Additionally, women may experience a lack of feeling or numbness in previously erogenous zones, coital pain, and overall reduced sexual pleasure (Perz et al., 2014; Ussher et al., 2012). The cumulative impact of these changes can lead to negative body image (Parton et al., 2016), disembodiment, and feelings of decreased sexual attractiveness (Berterö & Chamberlain Wilmoth, 2007; Parton et al., 2017b), impaired femininity (Archibald et al., 2006), and depression and anxiety (Heyne et al., 2021).

There is increasing interest in how cancer may uniquely affect sex, intimacy, and sexual embodiment among lesbian, gay, bisexual, transand queer (LGBTQ) people (Berner et al., 2020; Denes et al., 2024).^1^ Cisgender lesbian and bisexual women experience increased rates of cancer compared to cisgender heterosexual women due to a higher prevalence of associated risk factors, as well as higher rates of cancer related distress and unmet needs (Kano et al., 2022; Meads & Moore, 2013; Quinn et al., 2020). There is some evidence that lesbian women experience less disruption to their sex lives after cancer diagnosis and treatment than heterosexual women (Arena et al., 2007; Boehmer et al., 2009; Ussher et al., 2014; Winterich, 2003). This includes fewer problems with lubrication, achieving orgasm, and body image, and greater support and understanding between partners when compared to heterosexual women (Boehmer et al., 2009). While many lesbian couples do engage in penetrative sex (Roberts et al., 2000), it is less likely to be the focus of sexual interactions (Rothblum, 1994), as non-genital intimacy is a central part of the lesbian sexual repertoire (Ussher et al., 2014).

Little is known about the experiences of post-cancer embodiment and sexual well-being among trans people with a cervix. A scoping review of the sexuality of cancer patients from sexual and gender minority groups revealed a significant gap in research addressing the sexual concerns of this population within cancer care (Rodrigues et al., 2023). The embodied changes accompanying cancer can impact experiences of gender identity (Taylor & Bryson, 2016). For trans people, changes to embodiment resulting from cancer treatment may exacerbate gender dysphoria, or conversely, be experienced as gender affirming (Taylor & Bryson, 2016; Ussher et al., 2023b). A bilateral mastectomy is similar to gender-affirming “top surgery,” which may facilitate an alignment between masculine or non-binary gender identity and embodiment (Taylor & Bryson, 2016); however, a bilateral mastectomy may not achieve the aesthetic of a masculine chest contour. Some people embrace the choice to forego breast reconstruction after mastectomy, deciding to “go flat” (also known as “flattopping”) (Ussher et al., 2022a, 2023a). Conversely, mastectomy may be perceived to erase self-perceived femininity or reverse embodied changes that have occurred as part of gender affirmation, making breast reconstruction desirable (Brown & McElroy, 2018; Ussher et al., 2021, 2022a, 2023a). These embodied changes may impact the renegotiation of sex and intimacy following cancer. 

Positive body image contributes to better sexual functioning after breast cancer (Ganz et al., 1999), and lesbians have been shown to experience fewer body image concerns, and greater comfort being naked with others before and after treatment for cancer, compared to heterosexual women (Fobair et al., 2001). Bodily changes associated with cancer treatment, such as scarring, loss of hair or changes in weight have been reported to be experienced as abject by cis-heterosexual women with cancer (Parton et al., 2016). Abjection is a theory developed by Kristeva (1982) which explores the cultural positioning of the corporeality of women’s bodies as a sign of pollution, disgust, and as outside the boundaries of “acceptable” femininity. It has been used to theorize the embodied experiences of cancer and cancer treatment, where the cancerous body is constructed and experienced as abject (Parton et al., 2016; Waskul & van der Riet, 2002).

Embodiment in this context can be understood as “the experience of living in, perceiving, and experiencing the world from the very specific location of our bodies” (Tolman et al., 2014, p. 760). Bordo (1993) analyzes how ideology and popular culture discipline and shape actual bodies. Heteropatriarchy, she argues, constructs women’s bodies as deviant and other, in need of “improvement” and correction, based on abstract ideologies and distinctive cultural norms. In the context of breast cancer, this may be observed in the policing and reinforcing of cis-normative and hetero-feminine bodily ideals among LGBTQ people with a cervix; for example, the assumption that “appropriately” feminine bodies have two proportionate breasts, and moreover, that this form of embodiment is desirable to all women (Sledge, 2019). Similarly, Haug et al. (1987) explore the sexualization of women’s bodies from a Marxist feminist perspective. Tracing the complex interplay of social, political and cultural forces which produce a normative “femininity”, the authors argue that women are not passively socialized into the norms defining female sexuality but are active in the construction of a form of sexuality which they experience as oppressive. An awareness of this process, they argue, is a step toward liberation.

Sexuality is corporeal and relational; individuals experience their sexual selves in connection with others and within complex sociocultural settings (Chrisler & Johnston-Robledo, 2018a). Women’s bodies are frequently the subjects of sexualized or aesthetic scrutiny and evaluation. These assessments, marked by intersections of race, ethnicity, age and gender, are normalized within society, and made manifest publicly—through behaviors such as street harassment, derogatory labeling, and criticism of gender expression (Ussher et al., 2022d), and individually—through practices of self-objectification and self-policing (Butler, 1993; Fredrickson & Roberts, 1997). An awareness of this scrutiny means that many women find it challenging to experience a sense of embodied sexual comfort and self-assurance (Chrisler & Johnston-Robledo, 2018b). Chrisler et al. (2018a) defined “embodied sexuality” as the capacity to explore and express one’s sexuality with the necessary knowledge, skills, and confidence to achieve positive, fulfilling outcomes—such as sexual well-being, personal subjectivity, self-efficacy, and agency. An embodiment perspective on sexuality centers the lived body as both subject and object, while acknowledging the influence of social norms and contextual factors inscribed on the sexual body (Chrisler & Johnston-Robledo, 2018a).

Cancer and its treatment can greatly affect a person’s gendered and sexual embodiment (Gilbert et al., 2013). Until relatively recently, little social science research has questioned the role of the body, its impact on gendered and sexual relations and its significance to the study of social inequalities (Fahs & Swank, 2015). Focus has instead been directed toward the body as “medical enterprise, a collection of parts and processes,” as opposed to its social, sexual and political functioning (Fahs & Swank, 2015, p. 149). The study of the social function of embodiment highlights the tense relationship between the body and its biological and social contexts, particularly regarding its connection to sexual feelings, thoughts, and actions (Braun, 2005; Coy & Garner, 2012). This is evident in heterosexual women’s constructions of sexual embodiment in the context of cancer, where intrapsychic negotiation of sexual and gendered discourse, the materiality of embodied change, and relationship context influenced their sexual subjectivity (Parton et al., 2016, 2017a, 2017b). From this perspective, social discourses of sex and gender contribute to sexual experience, shaping the possibilities and constraints for embodied sexual practice and subjectivities following cancer. For instance, women for whom sex decreased after cancer took up a position of “embodying the absence of sexuality” and positioned their bodies as outside the “normal” hetero-feminine experiences of the sexual body (Parton et al., 2017b). Such accounts opposed the women’s reported experiences of embodiment before cancer and were evidence of a dysfunctional body remaining past treatment (Williams, 1996, 1998).

Previous research on cis-heterosexual women’s sexual well-being after cancer has focused on physical changes, where “functional” sexuality is narrowly conceived as heterosexual sexual intercourse (Fobair et al., 2006). This reflects the dominance of the “coital imperative,” which assumes heterosexual penetrative sex to be the only “normal” and “natural” form of sex; with other forms of sexual practices deemed to be secondary, supplementary or substitutional to the “real thing” (McPhillips et al., 2001). Adherence to the coital imperative has been linked to difficulties renegotiating sex and intimacy after cancer in cisgender heterosexual people (Parton et al., 2017b; Ussher et al., 2013). Many health care professionals (HCPs) currently lack the knowledge and education required to meet the needs and concerns of LGBTQ cancer patients and display cis-heteronormative biases in the health-care setting (Alpert et al., 2020; Tamargo et al., 2022). As a hegemonic construction of sex, the coital imperative has the potential to influence HCP perspectives and as such may impact the treatment options, information and support made available to LGBTQ people with cancer and their partners (Ussher et al., 2013). It is therefore necessary to consider the extent to which such frameworks can address the sexual concerns and needs of LGBTQ people with a cervix and their partners.

It is well documented that intimate partners—whom most often are also the carers of people with cancer—desire communication, information and support about post-cancer sexual changes to their own sexual selves and their relationship (Gilbert et al., 2009, 2016; Stewart et al., 2021). There is some evidence that partners may have a greater need for information than people with cancer (Laryionava et al., 2018; Rees & Bath, 2000). People with cancer have reported that it is important for their intimate partner to be included in discussions about sexuality with HCPs (Flynn et al., 2012), with open communication between people with cancer and their partner central to managing changes to sexuality (Badr & Carmack Taylor, 2009; Liberacka-Dwojak et al., 2023).

Previous research on LGBTQ people with a cervix has largely focused on sexual embodiment among cisgender lesbian and bisexual women with breast or colorectal cancer (Lisy et al., 2018; Rodrigues et al., 2023; Taylor & Bryson, 2016). Experiences across broader LGBTQ communities and tumor types, are lacking and require closer attention (Alpert et al., 2021; Pratt-Chapman et al., 2021; Quinn et al., 2015). Research focused solely on cis-heterosexual women in the context of sexual and reproductive health ignores the needs and experiences of trans and non-binary people with a cervix and may lead to data that is irrelevant or incorrect for this population. Trans and non-binary people with a cervix may have overlapping—but also unique experiences regarding sexual and reproductive health compared to cis-heterosexual gender women (Moseson et al., 2020). LGBTQ people with cancer and their partners have unique cancer experiences and needs related to their sexuality and gender identities, which often go unaddressed due to assumptions of heterosexuality and cisgender normativity in cancer care (Lisy et al., 2018; Quinn et al., 2020). This has implications for LGBTQ people’s access to culturally safe, relevant and tailored support, information and resources; the surgical and treatment options made available to them, their sexual embodiment; and their negotiation of sex after cancer treatment (Brown & McElroy, 2018; Taylor & Bryson, 2016). Addressing these gaps is a significant quality of life issue (Ussher et al., 2022c).

This paper considers the sexual and gendered embodiment of LGBTQ people with a cervix in the context of cancer and cancer caring, highlighting their unique embodied experiences and needs, with the aim of informing LGBTQ culturally safe and inclusive cancer care. The research questions shaping this paper are: (1) What are the subjective experiences of LGBTQ people’s embodied sexual changes and sexual renegotiation during and after cancer? (2) What are the implications of sexuality and gender diversity for experiences of sexual renegotiation and access to relevant, tailored cancer information and resources?

## Method

### Participants and Procedure

This qualitative study is part of the larger mixed method “Out with Cancer Study” that explores lesbian, gay, bisexual, trans, queer and intersex (LGBTQI) experiences of cancer survivorship and cancer care from the perspectives of people with cancer, carers and HCPs (Allison et al., 2024; Power et al., 2022, 2024; Ussher et al., 2022b, 2022c, 2023b, 2023c, 2024). This paper reports on a subset of this data utilizing interview data with LGBTQ people with a cervix. An integrated knowledge translation (iKT) framework (Graham et al., 2006) guided the study, comprised of a 46-member stakeholder group of LGBTQI and cancer organizations, and LGBTQI people with cancer and their carers who acted as collaborators throughout the design, data collection and analysis of the study.

Participants were recruited through cancer and LGBTQI community organizations, including the study partner organizations, social media (Facebook, Twitter, Instagram), cancer research databases, cancer support groups and LGBTQI community events. Snowball sampling was also used, asking participants to pass the study information to someone they knew who fitted the study criteria. The study was open internationally, although recruitment focused on Australia and other English-speaking countries such as the USA, UK, New Zealand, and Canada. Participant demographics were monitored, and recruitment strategies were refined through the data collection period with the aim of increasing the recruitment of underrepresented groups. Recruitment was open from September 2019 to September 2021.

The identifier “LGBTQ people with a cervix” (hereafter referred to in the text as LGBTQ people) is used to describe cisgender, trans and non-binary people with a cervix. A total of 57 LGBTQ people and 14 partners including a range of age and tumor types completed a 60-min semi-structured interview. The most common participants were lesbians with breast cancer, followed by lesbians with uterine cancer, as well as partners to participants with ovarian cancer. Most participants were cisgender, white, older adults, living in Australia. Nine people with cancer were trans, as were three partners (Table 1).

**Table 1:** Characteristics of LGBTQ people with cancer and partners

Demographic/personal characteristic	LGBTQ person with cancer (*n* = 57)	Partner (*n* = 14)
	Mean (SD), range	Mean (SD), range
Age at time of the study (years)	52.9 (14.5), 25–92	56.7 (12.2), 32–76
	*n (%)*	*n (%)*
*Sexuality*		
Lesbian, Gay	43 (75.4%)	9 (64.3%)
Bisexual	4 (7.0%)	0
Queer	6 (10.5%)	3 (21.4%)
Different or unclear identity	4 (7.0%)	2 (14.3%)
*Gender*^a^		
Female	48 (84.2%)	11 (78.6%)
Male	2 (3.5%)	1 (7.1%)
Non-binary	6 (10.5%)	2 (14.3%)
Other trans identity	1 (1.8%)	0
*Cancer type*^b^		
Breast	17 (29.8%)	6 (42.9%)
Colorectal	3 (5.3%)	0
Head and/or neck	2 (3.5%)	0
Leukemia	1 (1.8%)	1 (7.1%)
Lung	1 (1.8%)	0
Lymphoma	3 (5.3%)	0
Skin	4 (7.0%)	1 (7.1%)
Uterine, ovarian or endometrial	12 (21.0%)	3 (21.4%)
Something else	8 (14.1%)	3 (21.4%)
Not sure or unknown	6 (10.5%)	0
*Ethnicity*		
White	45 (78.9%)	11 (78.6%)
Asian	2 (3.5%)	0
Aboriginal, Torres Strait Islander or Māori	2 (3.5%)	1 (7.1%)
Mixed background	3 (5.3%)	0
Different or unclear background	5 (8.8%)	2 (14.3%)
*Location*		
Urban	28 (49.1%)	10 (71.4%)
Regional	17 (29.8%)	2 (14.3%)
Rural or remote	9 (15.8%)	1 (7.1%)
Not stated	3 (5.3%)	1 (7.1%)

^a^Sex assigned at birth for all participants was female

bCancer type for carers is of person cared for

### Measures

Semi-structured, one-to-one interviews in a conversational style were undertaken to explore subjective experiences of cancer and cancer care in-depth. The interview schedule asked open-ended questions such as “Can you talk to me about the impact that cancer has had on your: intimate relationships/sexual well-being/feelings about your body sexual identity/gender experience or expression? Questions pertaining to access to relevant care included probes such as: “Tell me about your experiences finding information or resources useful to you as a [LGBTQI + person] with [cancer type]?”, and “Can you tell me about your experiences of care provided by health professionals in relation to you being [LGBTQI +] and having [cancer type]? Throughout the interview process, the line of questioning was not always unilateral and participants would at times direct the topic of conversation. As qualitative interviews are by nature focused on the participant’s worldview and “frame of meaning,” it was fitting to remain adaptive, responsive, and be guided by issues of relevance to the participant (Nathan et al., 2019).

Audio-recorded interviews were conducted over the telephone or using the video-conferencing program, Zoom. While the broader study focused on cancer and cancer care, this study centered on questions relevant to experiences of sex and intimacy. These questions covered topics of embodied sexuality, sexual changes and renegotiation in the context of cancer, changes to relationships, as well as HCP interactions and support surrounding these topics.

### Data Analysis

Reflexive thematic analysis was used to analyze the interview data. This form of analysis identifies themes, or common stories, across the data and then considers patterns and subjectivity in participants accounts relevant to those themes (Braun & Clarke, 2019). Here, themes are guided inductively from the data rather than derived from existing theory. Braun and Clarke (2019) have emphasized the importance of reflexivity to thematic analysis, wherein the themes developed are not simply waiting to be “discovered” in the data, but are “creative and interpretive stories about the data, produced at the intersection of the researcher’s theoretical assumptions, their analytic resources and skill, and the data themselves.” These stories are always context-driven, situated, and the result of prolonged data immersion, consideration and reflection—an active and generative process.

This analytic approach required the data be coded and organized according to the patterns identified within the interviews. To become familiar with the data, a random subset of participant transcripts was closely read, individually and in collaboration with the stakeholder group made up of partner organizations, chief investigators, and community stakeholders. Individual notes were made to capture relevant concepts or “codes” present in the data. These first-order codes were mostly descriptive and reflected the semantic content of the data (Braun & Clarke, 2006). Some examples of first-order codes are concepts such as “avoiding sex and intimacy,” “vaginal changes,” and “partner/ex-partner support.” Through a process of discussion and decision-making with the research team, first-order codes were then grouped where commonalities occurred, making a smaller number of more distinct categories. We also added overarching codes, such as “sexual changes and renegotiation,” “sexuality and gender identity,” and “support in relation to cancer, being LGBTQI (positive/negative)” under which first-order codes sat. Following development of the coding framework, transcripts were imported into NVivo and analyzed using reflexive thematic analysis. Through iterative review and data comparison of the coding framework, themes in the data were generated. Each theme was assigned a distinct name to capture its essence. The themes and direct quotes from participants are presented below. When referred to in the text, participants are represented as: Pseudonym (Age, Partner, Sexual orientation, Gender, Cancer type). CF: Cisgender female; LGBTQ: Lesbian, gay, bisexual, trans, queer; NB: Non-binary.

#### Reflexivity Statement

Throughout our analysis, we engaged in a process of reflexivity. This involved being aware of the interactions between the researcher and participants and critically reflecting on how our own social and cultural backgrounds, assumptions, and positions influenced the research process (Finlay & Gough, 2003). The broader research team and advisory group included individuals of diverse gender and sexual identities; both those who have experienced cancer and those who have not; and members from both academic and clinical environments and community organizations. The authors of this paper acknowledge their various identities—as white, middle class, western, queer and non-queer, cis-gendered women researchers who have not experienced cancer—invariably shape the lens with which we bring to the analysis. Although the disparities between researcher and researched may be lessened due to a shared investment in or experience of culture, identification, or history, this in no way offered an exhaustive understanding of the research participants experiences. In this vein, we continuously discussed throughout the research process how our own positionalities may have shaped the way in which we viewed accounts in the data, ensuring that we prioritized and amplified the voices of the women in the study.

## Results

Participants reported changes to their sexual embodiment during and after cancer treatment that impacted their libido, sex practices, and relationships with partners in varied and complex ways. These changes are reflected in the following three themes: “LGBTQ people’s experiences of abject embodiment after cancer”; “HCP regulation of LGBTQ people’s bodies”; and “Sexual renegotiation and coping.”

### “Damaged,” “Broken,” and “Hideous”: LGBTQ People’s Experiences of Abject Embodiment After Cancer

While the majority of LGBTQ people reported material and intrapsychic changes to their sexual embodiment during and after cancer treatment, only some of these people positioned their bodies as abject—represented by the seven participant responses below. Constructions of abject embodiment affecting sex and intimacy were associated with cancer treatment impacts, including scarring, chronic pain resulting from mastectomy, incontinence, weight fluctuation and vaginal changes leading to a diminished sex drive. These constructions can be categorized into two subthemes: breaching bodily boundaries and avoiding sexual intimacy.

### Breaching Bodily Boundaries

Myra (68, Lesbian, CF, Breast) described difficulties having sex with her partner since receiving a partial mastectomy on her left breast. Prior to surgery, Myra’s breasts were a significant part of her sexual embodiment and sex practices—“there was so much fun to it.” Since surgery, the remaining pain, “a burn that will not stop,” has served as a reminder of “this poor little breast, what it’s got done to it. It doesn’t look right, it doesn’t feel right.” Due to this, Myra explained:

It was hard to see [them] as anything but damaged, broken. My left breast never again will be a sexual thing, ever, never be. It does have a big impact on you… Look, the thing about cancer is you really want to forget it, but my breast has never stopped hurting.

In this account, Myra positions her left breast as a site of deficit and abjection, which persists long after the end of treatment due to chronic pain. Similarly, Myra must live with “the visual legacy” of breast cancer. Specifically, the pain, distortion and asymmetry of Myra’s chest served as a reminder of the relationship between body, self-image, gender identity and sexual expression. This is reflective of cancer’s liminality, the ambiguity and uncertainty often experienced by people impacted by cancer (Blows et al., 2012).

Other participants reported experiences of abject embodiment, including bodies that were “tainted,” “maimed,” “hideous,” or a “no go zone.” When Leslie’s (32, Partner, Queer, NB, Breast) partner was diagnosed with breast cancer, she “felt like something had invaded her body and she just couldn’t get past it. Her breasts were always something that’s a really big part of her intimate relationships. Now it’s become *tainted* by this disease.” Christine (53, Lesbian, CF, Multiple), who has a hysterectomy scar traveling from her chest to her pelvic bone, explained “I have to say I’m not a very vain person, but I mean, definitely you feel your body is a bit maimed from it.”

After receiving a stoma surgery, Riley (53, Lesbian, CF, Ovarian) became acutely aware of her identity as “a cancer patient,” which in turn impacted her sex life. Riley described herself as “normally, not hugely self-conscious body image-wise” and as not “buy[ing] into the whole advertising, social media, you know, that kind of stuff that makes women feel like shit.” Nevertheless, Riley struggled to resume an intimate relationship with her partner while “having a bag full of shit on my stomach.” As she explained:

My partner helped me change it. I had trouble with that. And you know, she’d kind of been put on all the grotty jobs like giving me enemas and all of that kind of stuff. And the last thing, I felt was—I didn’t actually feel like having sex.

For Riley, the materiality of the ostomy bag is identified as breaching the body’s boundaries, intervening in her desire for sex; she positions her body as asexual and ultimately, abject. Sabrina (54, Lesbian, CF, Ovarian) who also had “bile and bowel issues” after surgery for ovarian cancer explained, “the bowel stuff is pretty hideous. I’m lucky the signaling [signals between the colon and the brain] is there, but I wasn’t sure it was for a while. All of that has made me feel pretty weird about my own body and myself as a sexual being.” Joyce (47, Lesbian, CF, Esophagus), attributed disturbances to her sexual embodiment to weight loss–“almost 60 kilos,” as well as her partner having seen her “vomit all that time” during treatment:

I did feel different and unattractive because I had, like, saggy skin. You just, you feel so self-conscious it’s hard to put yourself into that mindset, I guess... [Partner name]’s got the patience of a saint. She doesn’t see me any differently from what she saw before. I often said to her how can you even look at me sideways, like, you had to watch me vomit all that time. It was not good.

Due to the impacts of chemotherapy treatment, Jasper (50, Queer, CF, Breast) felt that her vagina was “broken”:

I literally could not touch myself at all without my skin just shredding. So that puts you off any kind of sexual activity, be it [with your] self or with someone else... my crotch turned into a ‘no go’ zone. I think the psychological effect of that was that it’s broken down there and it’ll never be fixed.

Albeit in different ways, Myra, Christine, Leslie, Riley, Sabrina, Joyce, and Jasper each indicated an acute “awareness of the proximity of sites of pleasure and excretion,” which has been shown to be common in individuals with chronic illness, particularly those with bowel dysfunction (Manderson, 2005). This awareness impacted on participant’s sexual embodiment in that they struggled to sexualize a body that was at once a defecating, saggy, and vomiting body—images that in most contexts are considered irreconcilable. It is those physical breaches of the clearly defined “inside/outside” bodily boundaries that hold the potential to strip intimate moments of their sensual pleasure (Ehlers & Krupar, 2022). This bodily transgression has the capacity to infringe upon LGBTQ people’s sexual relationships, and signals the need to preserve or reconstruct a sense of bodily integrity and aesthetics when re-establishing embodiment after cancer treatment (Ehlers & Krupar, 2022).

### Sentenced to the Sexual “Dead Zone”: Avoiding Sexual Intimacy

LGBTQ people’s discussions of libidinal changes were at times accompanied by metaphors of death and loss, where they positioned their bodies as barren and unfeeling. For example, Virginia (48, Lesbian, CF, Lymphoma) explained, “I was sentenced to early menopause and lost all sex drive. I just didn’t really use that part of my body. It was almost like a bit of a dead zone really. There was just no urge.” Virginia constructs early menopause as a punitive “sentencing” resulting from chemotherapy. Myra (68, Lesbian, Breast) explained that while non-genital intimacy remained present in her relationship–“we’re always touchy, we’re feely and we cuddle. We say we love each other”–sex had “absolutely stopped cold stone-cold dead, any sexual intimacy, for quite some time.” Sandra (70, Partner, Lesbian, CF, Breast) also drew on metaphors of death when reflecting on her partner’s response to chemotherapy, stating “the thing about the chemo is it kill[ed] all the sexual feelings that she had... It didn’t stop me loving her. [The chemo] killed it [partner’s sex drive] absolutely.” Despite Sandra’s reliance on metaphors of death to recount her partner’s libido loss, she later positioned sex as secondary to survival, where “being together” was considered more important than maintaining sexual intimacy. Similarly, Tracy (52, Partner, Lesbian, CF, Breast) stated of her partner with breast cancer, “Yes I’d like us to have sex more often but it’s just not up there on the things I truly care about.” Cancer, like the abject, was in effect a sense of “death infecting life” (Kristeva, 1982, p. 4).

### The Coital Imperative: HCP Regulation of LGBTQ People’s Bodies

Most participants in this study who experienced sexual changes expressed their desire to have conversations with HCPs about their sexual concerns and intimacy that reflected their experiences as LGBTQ people. Participants commonly reported medical conversations to be inadequate and overly focused on coital and penetrative sex practices.

Ash (40, Bisexual, NB, unknown), for example, found the information and resources made available to them after treatment to be “vague,” “dismissive” and focused on “penis and vagina issues” at the expense of a “range of [other] potential [sexual] activity and concerns.” Flynn (34, Queer, NB, Uterine) noted “it was really clear that in my gynecologist’s mind sex is penis and vagina penetration,” and Jasper (50, Queer, CF, Breast) emphasized the need to engage in “realistic conversations about sex rather than basing all conversations around penis-in-vagina sex,” concluding that “maybe we can talk about more ways to be intimate than whether or not you can still have PIV [penis-in-vagina sex].”

Catherine’s (46, Bisexual, CF, Vulval) treatment for vulval cancer required undergoing a radical vulvectomy, including the removal of her clitoris which resulted in significant changes to her sexual functioning, such as numbness to her vulva. Only one of Catherine’s HCPs understood the importance of clitoral stimulation to her sex life, and as a result, she was “put in a position of explaining to all my health professionals (except my oncologist) how my sex life actually worked before cancer and why I was so frightened that I might never orgasm again.” When Catherine raised concerns over whether she would be able to reach orgasm post-treatment, she was reassured by HCPs that her vagina would remain intact and that she was “lucky to be alive.” This led Catherine to feel unheard, as she explained:

Everyone’s going “Oh but you’ll still be alive. That’s what you need to focus on.” No, I want to know what my quality of life is going to be. If I’m still going to have the life that I’ve had pre cancer…there seemed to be a lot of ignorance among medical practitioners about what a lesbian sex life involves. To them removing my clitoris was nothing. They couldn’t understand why I was worried about that when their focus was the fact that my vagina would still be operational, so everything is going to be fine.

Having to repeatedly educate HCPs on the function of clitoral stimulation to her sexual pleasure led Catherine to feel “embarrassed” and “sick and tired.” She described this labor as feeling that she had become “the poster girl for lesbianism” in her cancer care interactions. She continued,

It was as if they had never met a gay woman before … never had any information about a lesbian sex life outside of the classic pornographic portrayal of [lesbians] … I should be able to put things in my vagina so what was my problem?

Sexual functioning is an important indicator of quality of life, and the implication here that an “operational”—in this context, penetrable—vagina is more important than one’s capacity to experience sexual pleasure is a heteronormative sexual script derived from the coital imperative and reinforced through biomedical discourses of HCP expertise (Gilbert et al., 2016; Ussher et al., 2013). As such, clitoral stimulation is positioned as merely a prelude to coitus or “real sex” (McPhillips et al., 2001; Parton et al., 2017b). Catherine’s experience of vulval cancer, a condition that is already “not seen,” “not heard of,” and “not talked about” is compounded by the lack of awareness and understanding of non-coital sex practices in cancer care.

A cis-heterocentric and phallocentric model of sex was also evident in the routine insistence from many HCPs on LGBTQ people’s use of vaginal dilators after pelvic radiation, to dilate the vagina to make coital sex and pelvic examinations more comfortable. This was often done without consultation regarding participants’ sexual practices, causing them to feel a sense of invisibility, discomfort and in some cases grief. For instance, Hannah, (45, Partner, Lesbian, CF, Uterine), referring to her partner with cancer, stated:

The only thing that they said was about [partner’s name] using those dilators to keep her vaginal vault open…that’s what caused a lot of grief because she was like, okay, I’ve been told by a man to put these dilators in me to increase a hole in me so that they can examine me or I can put a penis inside me. There was nothing about our relationship at all.

It was recommended that Miriam (51, Bisexual, CF, Multiple) used a dilator to mitigate scarring from one of her surgeries. She relayed, “I’m sure the doctors assumed that I was heterosexual because he said, ‘If you’re having regular intercourse, you don’t need to do this.’ So there’s an assumption on his part there…there was no discussion about my sexuality and any issues that that scar tissue could lead to.” Patricia (65, Lesbian, CF, Uterine) conversations with HCPs regarding her recovery from uterine “sounded like they were basing their whole understanding of what my sexuality was on some kind of penetrative heterosexual sex that was destined to provide pleasure to the partner and not your own pleasure.”

Barbara (48, Lesbian, CF, Uterine), similarly felt that her sexual needs and embodiment as a lesbian woman were neglected due to assumptions of penetrative sex from HCPs. She stated:

Every six months, I’m told, to maintain this vault. And every six months, I just have to present back at the hospital so that someone can inspect this vault. Did anyone ask me if I was going to use this vault, you know. Well, you know, maybe I don’t use my body in that way, you know? That would be my choice. But no one asked me.

Barbara described feeling “anger” at this omission, and the lack of relevant information available to meet her sexual concerns. This was exacerbated when Barbara attended a “second or third follow-up” with a specialist who she had previously informed of her sexuality, yet who still asked her to discuss treatment symptoms with her husband. She explained, “It was clear that he didn’t see me. He didn’t hear me. He didn’t understand who I was. That felt really difficult because obviously you’re in such a vulnerable position.” Barbara took to her art practice of ceramics to reproduce these dilators, which she described as “really helpful to take the power away from those objects... a reminder of that treatment, which was incredibly traumatic.”

Overall, participants’ experiences signaled to the dominance of heterosexism in cancer care—particularly in the treatment of reproductive cancers, which is reinforced through HCP expertise, and positions sex within restrictive, coitally focused constructions of (hetero)sexual functioning (Ussher et al., 2013).

### Sexual Renegotiation and Coping: Re-Imagining Intimate Relationships After Cancer

A significant proportion of the participants in this study reported renegotiating sex or intimacy within their relationships. For many, sexual renegotiation and coping after cancer involved practicing open communication, the exploration of new non-coital or non-penetrative sexual practices, and the incorporation of humor and creativity into their intimate lives. In most cases, cancer was an opportunity to fortify relationships, while relationship breakdown was less common.

For Catherine, speaking openly with her wife was crucial to regaining vulval and vaginal sensation after the loss of her clitoris, although she explained this to be an ongoing process:

My wife and I are just constantly talking to each other about [sex], because things are different obviously. I have some areas of my body that are just completely numb because of the nerve damage. Then, there are other areas that have been numb for the last few years that I’m starting to regain sensation. So it’s this constantly evolving process of just checking out what[’s] working now and what[’s] not. Does this hurt? Is that okay? Can you feel that? Just going with it from there.

Catherine’s wife’s unwavering support, acceptance, and emotional and physical “constancy” helped Catherine to “come to terms” with her post-treatment body. Catherine’s ability to communicate openly with her partner about her bodily changes, sexual needs, and concerns enabled a positive renegotiation of their sexual relationship. Once cleared by HCPs to “safely have an orgasm,” Flynn’s (34, Queer, NB, Uterine) ability to openly communicate with their partner enabled them to “climax.” They explained, “[Partner’s name] is a great partner; he listens to all of my instructions and was very responsive. It was really meaningful to have that kind of intimacy back.” Luca (33, NB, Queer, Bladder), who described themselves as “a bit too honest at times” emphasized the importance of relaying information provided by HCPs back to their partner in order to cope and “to know what to expect.”

In some instances, renegotiating sex and intimacy involved changing sex practices, and in others, foregrounding other non-genital forms of intimacy such as kissing, cuddling, and naked embrace were successful. Jasper (50, Queer, CF, Breast), for instance, described “taking it slow” with a new lover, despite her usual impulse “to take control” in sexual relationships. She recalled:

The first few dates were just kissing. Which was great…back to basics and it has been a little bit like a teenager... there’s plenty of time to get down to the toy draw... How good is kissing though. It’s really good. It’s like oh it’s like a lightning bolt through our tongues.

For Barbara, the difficulties brought forth by cancer enabled a new kind of closeness not centered on sex. She explained, “it’s changed how I feel about sex completely” in that “it just isn’t really on the radar.” This “doesn’t mean you don’t have physical intimacy with your partner” But that “[it] has changed.” Barbara commented, “I hate to say that difficult experiences bring people even closer together, but that is what’s happened.”

Martha (49, Lesbian, CF, Bowel) and her partner of 16 years described partaking in “other ways to have sexual intimacy” that do not “have to be a hard-core vaginal penetration” since the onset of cancer. Receiving pelvic radiation for lower rectal cancer had caused Martha to have major burns throughout her pelvis for three months, as well as long-term scarring in this area, including vaginally. Despite the repercussions from treatment, Martha and her partner had “had more of an intimate year than we’ve had in a long time,” partly due to the cancer-induced reprieve from “the thick of this parenting, full time work life.” In addition to enjoying non-genital intimacy, Martha and her partner found alternatives to non-penetrative sex. As she stated, “I’ve still got a working clitoris so we can still work around [it].” This adaptability and openness to forms of non-penetrative sex demonstrates a repositioning of sex as *more than “*hard-core vaginal penetration.” Archer (59, Lesbian, CF, Bowel) and her partner, who both received cancer diagnoses, similarly explored forms of non-penetrative sex. Archer explained that although cancer put “immense pressure” on the relationship, it was never “threatened” due to them being a “solid unit.” At times sex had to be reprioritized to “way down the list,” and at others, she explained “I was quite keen for sex... we’d just wade through it.” “Wading” through these periods of recovery and libido fluctuation involved finding alternatives to penetrative sex, such as forms of mutual masturbation.

Humor and creativity were key strategies in participants’ experiences of coping throughout cancer. Sabrina overcame challenges related to her sexual embodiment by incorporating humor and playfulness to her routine. Sabrina’s partner, Claire (65, Partner, Lesbian, CF, Ovarian), invented “Friday night dress-ups”—a weekly drag king show and “ritual” in which she would perform while Sabrina relaxed in their outdoor bathtub. “Drag kinging” is the practice of female-to-male “cross-dressing” and performance (Hanson, 2007). Sabrina recounted this with a smile in her voice:

Some nights, it was [Claire] in chaps and a hat, and our bath is outside, we light a fire underneath it. So, I’d be in the bath and [Claire] would come out dressed in all of the gear. So, you know, we had fun and she would make me laugh.

In adopting humor as a reparative tool, Sabrina and Claire managed barriers to sexual renegotiation and were able to redraw the boundaries of their own sexual embodiment after cancer treatment.

Despite challenges associated with cancer and cancer caring, including significant changes to the body such as scarring, chronic pain, incontinence, and weight fluctuation, relationship breakdown was less common among participants. However, in one account Christine’s (53, Lesbian, CF, Multiple) cancer diagnosis did lead to the dissolution of her sex life, and ultimately, her relationship. She explained:

At the beginning my partner was really supportive... But it turned out that she got really freaked out and actually didn’t want to touch me and she completely backed off me and our relationship totally got ruined and we broke up.

Since this experience, Christine has not dated “all that much” and is instead has focused on raising her child. Similarly, Ash (40, NB, Bi, Unknown) reflected “I had a partner who disappeared the day after I got the phone call that they’d found cancer.” Victor (47, Straight, TM, Ovarian), noted the effect of surgical treatment on his sex life, wherein “the nerves were so damaged and that impacted my relationship because I didn’t have a supportive partner.”

Other accounts attested to the potentially strengthening role of cancer on relationships. Flynn (34, NB, Queer, Uterine) noted:

Cancer could tear people apart easily. Cancer could make some people divorce, cancer could make some people stronger. But the way that we did it, we did it together and I don’t even know how to—I don’t know how to quantify this for you but we killed it, the [participant’s name] fucking killed it and we’re still killing it because it’s not done and it’s not going to be done. There’s screenings to be had and there’s a lot of emotional repair that needs to happen now and we’re going to kill that, too.

Cancer, for Flynn, served as a unique opportunity to “kill” cancer treatment, while for Cameron (Partner, 38, NB, Queer, Breast), cancer “solidified the possibility of getting married” to their partner.

## Discussion

This study explored the subjective experiences of LGBTQ people’s embodied sexual changes and sexual renegotiation during and after cancer, as well as the implications of sexuality and gender diversity for experiences of sexual renegotiation and access to relevant, tailored cancer information and resources. This research confirms prior findings that the impact of altered sexual embodiment due to cancer is seriously challenging for a large proportion of participants and their partners (Gilbert et al., 2010a; Parton et al., 2017a), and that in large, oncology HCPs currently lack the knowledge and education required to cater to the needs and concerns of LGBTQ cancer patients, and exhibit cis-heteronormative biases in the health-care setting (Ussher et al., 2022b, 2022e).

Disruptions to sexual embodiment led some of the participants in this study to experience their bodies as abject, as reported previously in research with cis-heterosexual women who have cancer (Parton et al., 2016), instilling in women a loss of control and feelings of uncertainty (Parton et al., 2017a). Discourses related to a loss of bodily control have been identified elsewhere, in response to embodied change constructed by cis-heterosexual women as “unfeminine,” “abnormal,” “strange,” or “grotesque” (Manderson, 2005; Parton et al., 2016; Waskul & van der Riet, 2002). Scarring may also serve as a distressing reminder of cancer’s potential recurrence or metastasis (Manderson & Stirling, 2007). Several participants drew on metaphors of death and loss in relation to libidinal changes, where they positioned their bodies as barren and unfeeling, and menopause as a punitive “sentencing” resulting from chemotherapy.

The participants in this study repeatedly expressed their desire to have medical conversations about their sexual concerns and intimacy after cancer that reflected their experiences as LGBTQ people. Open and non-judgmental communication and support, including sensitivity to the unique meaning of treatment-induced changes to LGBTQI people—including sexual embodiment—has been shown as central to HCP inclusive and reflective practice (Pratt-Chapman et al., 2022a, 2022b; Quinn et al., 2020; Ussher et al., 2022b). However, due to the prevalence of cis-heterocentric constructions of sexuality in healthcare and biomedical discourses of HCP expertise, participants’ concerns regarding non-heterosexual sex practices were seen as largely unimportant.

Assumptions by HCPs of penetrative and/or heterosexual sex, reinforced by the coital imperative, are widely reported across the literature on LGBTQ cancer care (Ussher et al., 2013). These assumptions were harmful to participants at multiple levels; they noted feelings of grief, anger and of being an outsider in their cancer treatment and did not feel they were equipped with the appropriate knowledge and information to address their experiences. In addition, HCPs often overlooked non-penetrative sexual practices such as oral sex and mutual masturbation and positioned digital penetration as secondary or supplementary to heterosexual penetrative sex. Studies have shown that lesbians may be more likely to engage in “autosexual” activities (Burleson et al., 2002), as they are outside of the heterosexual matrix and do not define sex as coital. Indeed, greater comfort with masturbation may facilitate adjustment to a body altered by cancer treatment in that it can enhance awareness regarding the location and intensity of stimulation necessary to increase sexual pleasure (Heima & Meston, 1997). As with previous research, participants’ engagement in forms of intimacy such as mutual masturbation, were associated with couples’ positive renegotiation of sexual intimacy after cancer (Gilbert et al., 2010a).

What was not addressed during participants’ cancer treatment—the negative space of non-coital sex—is meaningful (Power et al., 2009). Social scripts construct expectations, norms and values that guide human behavior. They also structure social action by what is not “scripted” (Simon & Gagnon, 2003). The same can be said of sexual scripts. In this case, sexual HCP scripts that frame “real” sex as coital penetration construct an alternate sexual script by omission—if heterosexual penetrative sex is “real” then non-heterosexual and/or non-penetrative sex is unreal (Gavey et al., 1999). The dominant sexual scripts of cancer care are structured in a way that excludes LGBTQ identities and sexual practices, leaving participants to form sexual scripts within the absent spaces (Power et al., 2009). For instance, where the significance of the clitoris to sexual pleasure is misunderstood by HCPs. Procedures due to cancer, such as a radical vulvectomy wherein the clitoris is removed, can have enduring psychological, emotional, sexual and physical impacts on women of all ages (Barton, 2003; Jefferies & Clifford, 2011; Senn et al., 2011). Yet clitoral knowledge is often omitted from biomedical discourses, which can in part be explained by the reproductive bias of the anatomies of female and male genitalia (Tuana, 2004). That is, because medical knowledge regarding female sexual pleasure has historically focused on reproductive systems over so-called “external genitalia,” the clitoris and the labia have often been ignored in contemporary accounts of female anatomy (Tuana, 2006). Nevertheless, penetrative sex was not uncommon among participants; vulval pain was mitigated with the use of lubricant. Knowledge surrounding the renegotiation of penetrative sex in LGBTQ relationships affected by cancer is therefore an important point of education for HCPs.

Because some participants’ sexual practices were misunderstood by HCPs, their concerns about bodily changes, sex, and intimacy tended to be poorly addressed in resources and medical interactions. This had important material consequences for participants and their partners, leaving them ill-equipped to cope with changes to sexual functioning, feeling unsupported, distressed, and as though their sexual needs and concerns were of less importance than those of heterosexual patients. Relationship breakdown has been identified as an issue for couples in prior cancer research (Ussher et al., 2012), accompanied by increased conflict and communication issues (Badr & Taylor, 2006; Ussher et al., 2012; Zahlis & Shands, 1991). Participants in this study also showed strength in their relationships—citing cancer experience as a source of relational fortitude. Poor body image, sexual anxiety, and fear of negative responses to cancer status disclosure can lead women to avoid sexual relationships, remain in unfulfilling ones, or feel deprived of companionship, support, and intimacy (Hamilton & Zebrack, 2011).

However, participant’s accounts also illustrated great resilience in the face of embodied changes negotiating sex and intimacy after cancer. In the absence of relevant information and resources, they explored forms of non-genital intimacy, experimented with non-penetrative sexual practices, and talked openly about their needs. Communication between participants and their partners was key in facilitating sexual renegotiation after cancer, underlining research suggesting that communication is the strongest predictor of continued sexual activity after cancer (Perz et al., 2013, 2014; Ussher et al., 2015). Moreover, adopting a receptive approach to couple communication after cancer can help reduce distress and strengthen relationship satisfaction (Badr & Taylor, 2006; Badr et al., 2008; Manne et al., 2006). As such, HCPs can and should play a facilitating role in couples’ communication around sex in the context of cancer, and in empowering them to be sexually intimate following its onset. This could include education related to the effects of cancer and its treatments on sexuality and sexual functioning, and importantly, resources that normalize sexual alternatives to hegemonic discourses of sexuality that privilege the coital imperative.

## Conclusion

The findings of this study demonstrate a culturally and institutionally sanctioned cis-heterocentric construction of sexuality in oncological care, the implications of which suggest the cancer care needs of LGBTQ people and their partners are largely unmet. Acknowledging these needs would require a careful redressing of the “one-size-fits-all” approach common within cancer care (Banerjee et al., 2021; Shetty et al., 2016), such as in the training of HCPs in the practice of LGBTQ inclusive and affirmative cancer care. The provision of tailored information can empower LGBTQ people to define their own health needs, meanings, and values, rather than having these needs imposed upon them. It would involve recognizing the importance of the HCP-patient relationship in providing affirmative health care (Davies et al., 2021; Ussher et al., 2022b), and would value “communicative competence” (Banerjee et al., 2021; Quinn et al., 2020; Shetty et al., 2016; Webster & Drury-Smith, 2021). Practicing communicative competence would entail understanding the specificity of LGBTQ people as potentially vulnerable and needing nuanced information and care, following best practice models of person-centered care, which would include working in collaboration with patients and their partners, and being receptive to and respectful of their unique and subjective experiences (ACSQHC, 2019; Dunn et al., 2021).

Despite the myriad of challenges participants endured throughout cancer and its treatment, women creatively negotiated their experiences of sex and intimacy, demonstrating their resistance to cis-heteronormative social structures. It is critical we ask questions about the social, political, and medical responsibility of cancer care institutions, HCPs, and the discourses surrounding these spaces of culture, to take all embodied perspectives of cancer seriously—not only those represented by the heterosexual and cis-gendered majority. LGBTQ people’s stories offer critical insights for the development of meaningful and tailored information, resources, and support for this population moving forward. Their embodied experiences offer a more expansive, inclusive, and person-centered rethinking of survivorship—and sexual renegotiation—for all.

## Data Availability

The raw data supporting the conclusions of this article will be made available by the authors, without undue reservation.

## Footnotes

1 We use the term “trans” as an umbrella that refers to individuals who identify as transgender, gender diverse, or non-binary, as recommended by community partners and stakeholders on this project. In this study, the term non-binary refers to non-binary individuals with a cervix

## Code Availability

The coding framework supporting the conclusions of this article will be made available by the authors, without undue reservation.
